# Staffing remote rural areas in middle- and low-income countries: A literature review of attraction and retention

**DOI:** 10.1186/1472-6963-8-19

**Published:** 2008-01-23

**Authors:** Uta Lehmann, Marjolein Dieleman, Tim Martineau

**Affiliations:** 1 School of Public Health, University of the Western Cape, Bellville 7535, South Africa; 2 KIT Development Policy and Practice, Royal Tropical Institute, PO box 95001, 1090 HA Amsterdam, The Netherlands; 3 Liverpool School of Tropical Medicine, Pembroke Pl, Liverpool, L3 5QA, UK

## Abstract

**Background:**

Many countries in middle- and low-income countries today suffer from severe staff shortages and/or maldistribution of health personnel which has been aggravated more recently by the disintegration of health systems in low-income countries and by the global policy environment. One of the most damaging effects of severely weakened and under-resourced health systems is the difficulty they face in producing, recruiting, and retaining health professionals, particularly in remote areas. Low wages, poor working conditions, lack of supervision, lack of equipment and infrastructure as well as HIV and AIDS, all contribute to the flight of health care personnel from remote areas. In this global context of accelerating inequities health service policy makers and managers are searching for ways to improve the attraction and retention of staff in remote areas. But the development of appropriate strategies first requires an understanding of the factors which influence decisions to accept and/or stay in a remote post, particularly in the context of mid and low income countries (MLICS), and which strategies to improve attraction and retention are therefore likely to be successful. It is the aim of this review article to explore the links between attraction and retention factors and strategies, with a particular focus on the organisational diversity and location of decision-making.

**Methods:**

This is a narrative literature review which took an iterative approach to finding relevant literature. It focused on English-language material published between 1997 and 2007. The authors conducted Pubmed searches using a range of different search terms relating to attraction and retention of staff in remote areas. Furthermore, a number of relevant journals as well as unpublished literature were systematically searched. While the initial search included articles from high- middle- and low-income countries, the review focuses on middle- and low-income countries. About 600 papers were initially assessed and 55 eventually included in the review.

**Results:**

The authors argue that, although factors are multi-facetted and complex, strategies are usually not comprehensive and often limited to addressing a single or limited number of factors. They suggest that because of the complex interaction of factors impacting on attraction and retention, there is a strong argument to be made for bundles of interventions which include attention to living environments, working conditions and environments and development opportunities. They further explore the organisational location of decision-making related to retention issues and suggest that because promising strategies often lie beyond the scope of human resource directorates or ministries of health, planning and decision-making to improve retention requires multi-sectoral collaboration within and beyond government. The paper provides a simple framework for bringing the key decision-makers together to identify factors and develop multi-facetted comprehensive strategies.

**Conclusion:**

There are no set answers to the problem of attraction and retention. It is only through learning about what works in terms of fit between problem analysis and strategy and effective navigation through the politics of implementation that any headway will be made against the almost universal challenge of staffing health service in remote rural areas.

## Background

In recent years major initiatives have been launched to tackle health and inequalities in access to health. These include the Millennium Development Goals initiative and programmes to combat the major priority diseases (malaria, TB and HIV AIDS). However for these relatively well-financed initiatives to bear fruit, they require effective, efficient and equitably provided health services. The health workforce has been identified as the key to effective health services [1,2]. At the same time, however, shortages are the most commonly reported staff-related problem, especially in resource-poor countries [3,4].

**Figure 1:**
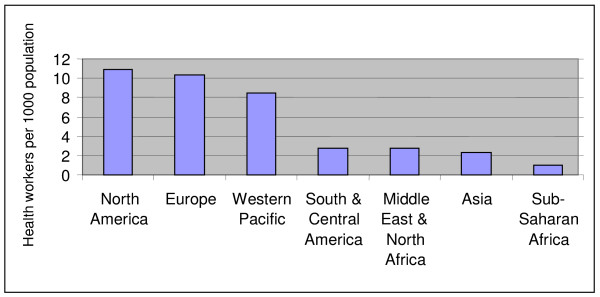
Health worker density by region. Source: JLI (2004), Fig. 1.7

The work conducted by the *Joint Learning Initiative *in recent years [1] has confirmed that global inequities in the distribution of health personnel hit those countries hardest which can least afford it. Asia, which has about half the world's population, has access to only about thirty percent of the world's health professionals. Africa, with a predominance of countries with severe shortages of health professionals, has the highest disease burden of any continent, thus underscoring the perverse relationship between need for healthcare and distribution of health personnel. The gap in sub-Saharan Africa is reflected by the distribution of doctors and nurses as shown in Figure 1.

The maldistribution of personnel has its roots in long-standing global inequalities. It has been aggravated more recently by the disintegration of health systems in low-income countries and by the global policy environment [1,5,6]. One of the most damaging effects of severely weakened and under-resourced health systems is the difficulty they face in producing, recruiting and retaining health professionals both in the country and in the health sector. Low wages, poor working conditions, lack of supervision, lack of equipment and infrastructure as well as HIV and AIDS, all contribute to the flight of health care personnel, a flight which has become a migration crisis for many low-income countries.

The inequitable distribution of health professionals found between countries is mirrored within countries. Most countries display serious disparities between levels of care and between urban and rural areas. In Bangladesh, for example, four metropolitan districts have 35% of all doctors but only 14.5% of the country's population (Hossain and Begum quoted in [7]. In Ghana, in 1997, 1087 of the 1247 (87.2%) general physicians worked in the urban regions, although 66% of the population lives in the rural areas [8]. The impact of this maldistribution on health care delivery in rural areas is profound, at times resulting in primary health care facilities being staffed solely by untrained staff [9,10].

Padarath et al. argue that the migration flows of health personnel follow a hierarchy of wealth and result in a global conveyor belt of health personnel moving from the remote rural areas of low-income countries via urban areas and/or the private sector in these countries into under-served areas in high-income countries [11].

It is in this global context of accelerating inequities that health service policy makers and managers are searching for ways to improve the attraction and retention of staff in remote and rural areas. But the development of appropriate strategies first requires an understanding of the factors which influence decisions to accept and/or stay in a remote post, particularly in the context of mid and low income countries (MLICS), and which strategies to improve attraction and retention are therefore likely to be successful. It is the aim of this review article to explore the links between attraction and retention factors and strategies, with a particular focus on the organisational diversity and location of decision-making.

The discussion of attraction and retention factors and strategies falls within the broad scope Human Resource Management (HRM) as a strategic and coherent approach to managing staff of an organisation [12]. HRM covers all aspects of staff management, including the resourcing of staff through attraction and retention strategies. Ensuring coherence of these HRM strategies is complex, as illustrated by Buchan with reference to nurses: "The complex interaction of pay, job satisfaction, career prospects and non-work issues mean that there is no single solution to retaining and motivating nursing staff" [13]: 216). The success of strategies within a health sector will also depend on the socio-economic, political and institutional context and on the health labour market: availability of resources, management skills, influences exercised by key stake holders, political will or even sabotage by dissatisfied stakeholders can play decisive roles in the success of failure of strategies. Recognising the complex interplay of factors impacting on attraction and retention, [14] advocates the use of "bundles" of linked and coordinated HRM interventions.

This review article focuses on the lower end of the hierarchy of wealth described by Padarath and colleagues and picks up on Buchan's suggestions of complex interactions between factors impacting on attraction and retention of health workers in remote rural areas in within mid and low income countries and a consequent need for bundles of HRM strategies to improve the latter. We take this one step further by exploring the organisational location of the key decision-makers with particular reference to the public sector. Taking stock of the current knowledge in this area will hopefully contribute to the on-going learning process in this somewhat neglected area and support further research on how the remote rural areas can be better staffed with health workers in the future.

## Methods

To find out what is already known about attraction and retention a review of the current literature was conducted.

The majority of the literature on the factors behind geographical distribution of staffing originates from industrialised countries. We therefore had to draw substantially on that literature, but where possible we have used experiences from MLICs. We recognise that the MLIC literature also covers a wide range of situations related to income levels, geographic locations, indigenous labour markets and balance of type of employer, making it more challenging to draw conclusions of universal relevance.

Because of our desire to capture as much literature as possible focussing on MLIC, and to provide a textured and rich review of factors and strategies, we opted for a narrative rather than systematic review method, using an iterative approach to finding new literature.

### Search methods

Document searches were conducted in a number of ways. We conducted Pubmed searches using the following search terms: *staff retention*, *retention in rural areas, retention in low-income countries, turnover, medically underserved area, job satisfaction*. These terms were searched by themselves and in conjunction with professional classifications: *health workers, nurses, doctors, mid-level workers*. In addition, we conducted a systematic search of pertinent journals, including the *Australian Journal of Rural Health, Health Services Research, Human Resources for Health*, the *Journal of Rural Health*, and *Rural Remote Health*, and included unpublished literature (using authors' knowledge of the field). Lastly, we used a snow-balling approach to identifying further literature from the reference lists in relevant journal articles. Our search initially included articles focussing on high- and low-income countries, but was then honed down to focus on low-income countries. About 600 papers were initially assessed and 55 eventually included in the review.

## Results

### Conceptual Framework

The extent to which health workers can be attracted to and retained in remote areas depends on two interrelated aspects: the factors which contribute to health workers' decisions to accept and the stay in a remote post; and the strategies employed by governments to respond to such factors.

There are many different theories and models coming from different disciplines which attempt to categorise and explain the factors impacting on workforce mobility. Some of these, such as the Neoclassic Wage Theory, suggest that the choice is driven largely by financial motives [15] and by the probability of finding employment [16]. Behavioural theories, such as those developed by Maslow and Herzberg, show a more complex decision-making process regarding the movement of labour [17] with a particular emphasis on the importance of job satisfaction. In the recent literature on health workforce mobility relating both to international and internal migration, factors have commonly been categorised into "pull" and "push" factors (see for example [7,11,18]. "Pull" factors are identified as those which attract an individual to a new destination. These might include improved employment opportunities and/or career prospects, higher income, better living conditions or a more stimulating environment. "Push" factors are those which act to repel the individual from a location. They often mirror "pull" factors and might include loss of employment opportunity, low wages, poor living conditions, etc. [15].

**Figure 2:**
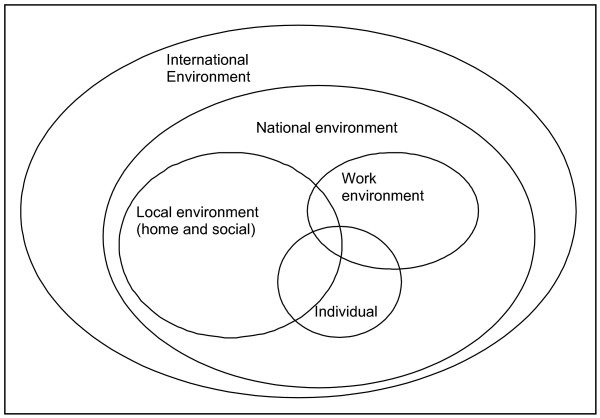
Different environments impacting attraction and retention.

Both push and pull factors impact on the individual who makes a decision about moving to, leaving or staying in a job in many different ways. Any decision by an individual will be the result of a complex interplay between these factors. For the purposes of analysis and strategy development it is helpful for policy-makers and managers to have some way of organising the different factors. We have done this by grouping the different types of environment surrounding the individual (see Figure 2).

The factors in the international environment are mainly pull factors such as higher salaries, better working conditions and better career opportunities in other countries.

The national environment comprises both push and pull factors such as the general political climate, including the degree of political and social stability, war, crime, etc., as well as general labour relations, the situation of the public service, salary levels, career opportunities, etc.

The local environment is primarily made up of general living conditions and the social environment.

The work environment again encompasses push and pull factors, such as local labour relations, management styles, existence or lack of leadership, opportunities for continuing education, availability of infrastructure, equipment and support.

Lastly, there are a number of individual factors which may impact on decisions, such as origin, age, gender and marital status. All factors will be discussed in more detail below.

**Figure 3:**
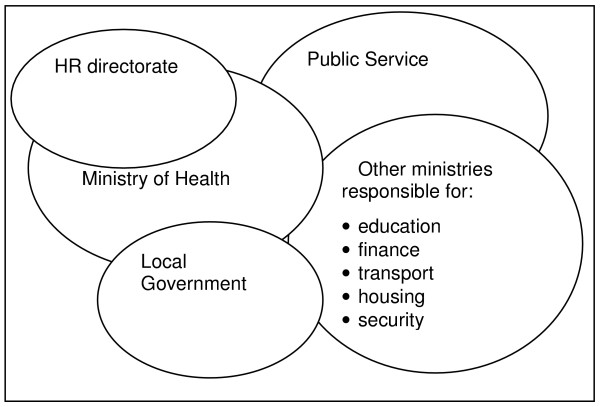
Location of decision-makers associated with attraction and retention in the public sector.

It is clear that employers will not have much influence beyond the work environment, though in the public sector the government can have more influence on both the local and national environment. In this review we focus on those factors which are potentially amenable to HRM strategies. To identify these factors it is important to identify the locations of structures involved in decision-making relating to employment and wider issues of attraction and retention. In the case of the public sector many decisions about HR policy and financing are made by central government ministries. The various locations of decision-making are illustrated in Figure 3.

How then do HRM strategies address the identified factors? Substantial work has been done in recent years, for example through the Joint Learning Initiative, to identify human resource measures to address key HR challenges [1,2]. But the documented examples of successful attraction and retention strategies in MLIC are limited. The above framework suggests that factors influencing individual choices, as well as strategies to be employed lie often in spheres beyond the immediate HRM or even health systems mandate. This raises questions as to how the development of strategies to improve attraction and retention should be developed and co-ordinated.

### Factors influencing attraction and retention in remote rural areas

This section summarises findings of the literature review on factors impacting on staff attraction and retention, with a focus on remote rural areas. While the factors are grouped under separate headings, it is important to note that they interact with and influence each other as reflected in Figure 2. Local working environments, for example, will be influenced and shaped by general living conditions as well as national policies, socio-economic status, etc. The complexities of interactions have been simplified here to better understand each factor.

#### Individual factors

Individual factors may depend on a person's personal characteristics, such as age, gender, marital status, etc. How they impact on an individual's decision-making is often fluid and may change in a person's life and career cycle.

The literature discussing personal and lifestyle factors focuses almost exclusively on high-income countries, although some literature from low-income countries is now emerging [19]. Much of it discusses generic considerations, not linked specifically to the health sector or to rural areas. In general, associations between demographic factors and reasons for leaving are inconclusive in relation to age, educational level, and gender [20].

With regard to marital status, studies showed no association with intention to leave work or decision to actually leave [21]. However, some researchers, quoted in Lexomboon, found that workers who were single indicated a greater intention to leave work and had higher turnover than married workers [22]. In Malaysia spouses were identified as having an influence on an individual's mobility, and family responsibility had more influence on female workers than on male workers [23]. While the mobility of men was found to be primarily related to economic considerations, the moves of women were closely related to marriage or family consideration.

Preferences of location may also depend on what kind of living conditions health personnel are used to. The correlation between geographical origin of students and their future choice of practice, i.e. whether students from under-served areas will return to under-served areas to practise their profession, is much debated in the literature. There is now considerable agreement in research conducted in high and low-income countries [22,24-27] that rural upbringing increases chances of health workers returning to practice in rural communities [28,29].

#### Local environment

While the literature is inconclusive on the role that individual characteristics play in choice of remote practice, it is quite unanimous that the general living environment, together with social obligations, are important elements in decisions on where to work.

Lack of housing, lack of health care and lack of schools for children are quoted internationally as reasons why staff either do not join or leave health services in remote areas. They were raised in research conducted among health care providers in the Navajo Area Indian Health Services [30], as well as in Ecuador [31]. The importance of general living conditions, including staff accommodation, schools and qualified teachers, good drinking water, electricity, roads and transport, also features very prominently in a study conducted by Mensah [19] into factors affecting retention in rural Ghana. When study participants were asked what factors would make them refuse postings, lack of staff accommodation was ranked first, followed by lack of schools and qualified teachers, good drinking water, electricity, roads and transport [19]. Participants were also asked what was keeping them in their posts (posting directive from above and to care for aging parents, featured most prominently), and what would attract them to remain in a so-called hardship area. Here again issues of general infrastructure were most common: good schools for children, accommodation, drinking water and a doubling of salaries.

#### Work-related factors

Reports in the literature differ on the importance of pay and conditions of service to a person's decision to choose a workplace. While salary was positively associated with decreasing intention to leave work of nurses in Thailand [32], WHO, in a study of reasons for staff mobility in six African countries, found that only 24% of respondents quoted better remuneration as a reason for leaving [18].

The pay issue is complicated, however, and some literature suggests that perhaps this factor should be broadened to cover the "ability to generate income". This might include what are sometimes referred to as 'coping strategies' such as a second job, theft, under-the-table payment or running a private practice in an urban area as a coping strategy to improve income levels [33]. In Angola, in the mid-1990s, for example, doctors could earn the equivalent of their weekly salary in the public sector in one hour of private work [34]. The implications of this are that the factors relating to the primary employment may be overridden by the availability of secondary employment, thus affecting people's choice of post and location

Vujicic et al., in a recent international study on the role of wages in the migration of health care personnel, found health care workers' willingness to migrate from a low-income to a high-income country "somewhat unresponsive to wage differences between source and destination countries" where the wage difference between source and destination country was between 3 and 15 times[35]. They cautioned, however, that practices might differ where wage differentials were higher.

Work environment and job satisfaction are other factors determining attraction and retention in high- and low-income countries [32,36,37]. A study among rural health workers in North Vietnam, for example, revealed that the most motivating factors in their job were identified as appreciation by managers, and colleagues, appreciation by the community, a stable job and income and training [38].

Working conditions, including organisational arrangements, management support, high-risk work environments and availability of equipment, have been identified by several authors as being a determining factor in deciding whether to leave or stay in remote areas [7,18,38-40].

The link between access to continuing education and professional advancement and retention is unclear. Much of the literature focussing on high-income countries did not find close correlations between opportunities of career advancement and turnover [41,42]. However, evidence from a six-country study in Africa (Cameroon, Ghana, Senegal, South Africa, Uganda and Zimbabwe [18], based on interviews with between 5 and 20% of the total number of skilled health personnel in the public sector in each country, showed a much stronger correlation. One of the main reasons for departure to a foreign destination in Ghana and Cameroon, for example, was the desire for further professional training.

#### National environment

The same six-country study [18] also emphasises the importance of the perceived national environment. It found that social unrest and conflict ranked high as a reason for emigration. For example, "Zimbabwean emigrants had emigrated because of economic reasons (55%), by far the most important push factor, followed by the decline of the health service (53%), lack of facilities (38%) and despair about the future of the country (38%)" [18].

An increasingly important factor is the impact that the presence of global health initiatives has on staffing in the public sector. Kushner et al. present anecdotal evidence of the brain drain into 'projects' in a letter to the Lancet [43]:

Staff at central and district health facilities are leaving in record numbers to work for programmes sponsored by either overseas universities or nongovernmental organisations. At LCH [*Lilongwe Central Hospital*], a 970-bed facility authorised to employ 520 nurses, currently only 169 nurses are available for clinical care. In the hospital laboratory, only six technicians are now working where 38 were previously employed. When asked where these workers are, the response is invariably the same – projects.

The exact impact of global health initiatives has not yet been sufficiently researched, although several research projects are presently under way. Indications are, however, that in some cases they may be weakening rather than strengthening the national health sector, particularly if issues of sustainability have not been sufficiently addressed.

#### International environment

The international context of dramatic and increasing health worker shortages in most high income countries plays an important role in shaping attraction and retention issues. Elements which act as pull factors to attract staff to international destinations include higher rates of remuneration, more satisfying working conditions, a safer working environment and better educational and career development opportunities, as well as broader factors such as higher quality of life, freedom from political persecution, freedom of speech and educational opportunities for children. While such pull factors play an important role, Padarath et al. argue, quoting a WHO study carried out in the late 1970s that "no matter how strong the pull factors are of the recipient countries, migration only seems to result if there are also strong push factors from the donor country" [11]. Their argument supports the suggestion that different factors interact in complex ways to generate an individual's decision regarding place of work.

The factors identified above re-enforce the view that HR directorates of ministries of health, or even the ministry itself, have a relatively limited scope to improve attraction and retention of health workers in remote rural areas. They may be able to bring some influence to bear on working conditions, including management styles, working environments and HR policy. However, many decision-makers who could develop and implement strategies to address attraction and retention are located outside the health sector. The development of a strategic and coherent HRM approach would therefore require multi-sectoral collaboration involving all the key decision-makers.

### Strategies to improve attraction and retention in remote rural areas

To address the problem of staff shortage in any situation there would logically be several broad options that policy makers can consider:

1. Address known attraction and retention factors for existing staffing types/structures.

2. Use different staff (usually less skilled) who are easier to attract and retain.

3. Change the nature of service delivery or, if necessary, stop providing it.

4. Develop alternative service delivery systems (e.g. partnerships with private sector, contracting out to private for profit or not-for-profit sector).

In this paper we limit ourselves to consideration of option 1, linked to the preceding analysis of factors affecting attraction and retention. The type of strategies for improving attraction to and retention in remote rural areas fall into four broad categories:

1. Recruitment and training for rural practice.

2. The use of incentives and compulsory services.

3. Improving working conditions.

4. Improving living conditions.

Documented strategies within these broad categories have been either employed individually or in bundles.

#### Recruitment and training for rural practice

Targeted recruitment and selection, the location and content of training programmes, as well as opportunities for continuing education are commonly used strategies.

The Government of Thailand, for example, has had considerable success in improving equitable access to healthcare throughout the country over the last four decades, and rural recruitment, in combination with rural location of training, has played an important role. Locally recruited and trained health workers are better equipped for their and prepared for living in remote areas. Nurses, midwives, junior sanitarians and other paramedics are recruited and trained locally, assigned to placements in their home towns and licensed to serve in the public sector alone [44,45].

In African countries a number of governments have taken steps to open new medical schools which introduce problem-solving, student focussed and community-based approaches to medical education. Examples can be found in Ethiopia, Ghana and Kenya [46]. However, to our knowledge the impact of these initiatives on short-term and long-term choice of practice has not been systematically evaluated, constituting an important knowledge gap and research requirement. A search of two relevant international journals, *Education for Health *and the *Journal of Rural and Remote Health *supports this finding. While an extensive literature exists which concerns itself with strategies to making health professions education more appropriate, very few studies report on the impact of educational changes on long-term professional choices and behaviours.

#### Use of incentives & and compulsory service

Countries have employed a number of different forms of incentives and compulsory service, either on their own or in combination to attract or compel health workers to work in rural areas.

Indonesia, for example, employed a combination of compulsory service, preferential access to training and financial incentives for working in remote areas. Doctors working in remote areas are able to earn double the salary of doctors working in urban areas, a difference, however, which is virtually eliminated by better additional earning opportunities in urban areas. They also increased their chances for being recruited into the prestigious civil service which provides state-subsidised specialist training. Preparedness to serve in remote areas increased with the introduction of this incentive, more substantially among graduates from schools from remoter areas, and less so among women with children [47].

In Thailand, students recruited by the Ministry of Public Health receive heavily subsidised tuition and free clothing, room and board, and learning materials during their studies in return for carrying out compulsory public health service – usually in remote areas – after graduation [44,45].

In South Africa the Ministry of Health introduced compulsory service as well as financial incentives to address inequities in the distribution of health personnel, the rural and scarce skills allowances. While the financial incentives appear to have influenced some health workers to change their short-term career plans, staffing of the most rural hospitals remains a problem, and hospitals in remote rural areas remain without doctors [48].

Zambia has recently introduced a package of measures to attract doctors to and retain them in remote rural areas [9]. The package includes a rural allowance equivalent to about 30 percent of their salary, but also the renovation of accommodation, contribution to school fees, vehicle and/or housing loans and some support for further education. The package appears to have an impact on attracting doctors who otherwise would not have gone to the rural postings. It was relatively expensive, but much cheaper than using expatriate doctors to fill the posts.

Many Latin American countries have made use of compulsory rural service, particularly for medical doctors, for many years. Mexico set up the first such programme in 1936. Cavender and Alban revisited the Ecuadorian programme, established in 1970, in the late 1990s [31]. They found that, although graduates felt ill-prepared for their rural service, given that they were largely trained in urban health problems, and although they faced numerous logistical problems, most appreciated the experience of working in remote areas. Unfortunately, the authors did not probe the correlation between the rural service experience and willingness to continue service in rural areas, although their study implies that this was not the case. They report on other authors' suggestion, however, that countries should consider the re-training of physicians to form rural health corps, given many Latin American countries' surplus of physicians.

Other writers argue that bonding and compulsory service arrangements are fraught with problems, as they are difficult to enforce, easy to undermine and most countries lack the administrative capacity and the political will to enforce such a system [48,49]. They point to the inter-relatedness of this and other measures to improve retention: their success or failure largely depends on the supportiveness of the broader system within which they are located.

#### Improving working conditions

There is very limited evidence of strategies aimed at improving working conditions and job satisfaction, although a number of studies discuss the benefits of introducing participatory management and flexibility in US institutions [39,50-52].

One exception is the introduction of supportive supervision, which has led to improved motivation in a number of countries, thus potentially impacting on decisions to stay. Case studies in a number of countries, including Papua New Guinea and the Phillippines found that supervision improved not only work satisfaction but also performance and quality of care in remote settings [53,54]. These results are supported by a six-country study which showed that quality of care improved by focussing on supporting performance of staff [55].

#### Improving living conditions

While the literature in many cases shows a correlation between quality of living conditions and willingness to move to or stay in a particular area, there is much less evidence in the literature that this knowledge has led to systematic and wide-spread efforts to improve living conditions in areas that struggle to attract or retain staff. An exception is Thailand which invested heavily in general rural infrastructure as part of its extensive district development programme: staff housing at rural district hospitals became a priority, as did basic infrastructure such as roads, phones, water supplies and radio communication. Nitayarumphong et al. acknowledge, however that despite these efforts, "living conditions in most of the rural districts are still very different from those of the central districts in terms of education, communication and transportation. This is still the limitation of health manpower distribution to rural areas [44]."

The Zambian Health Worker Retention Scheme – piloted initially for doctors – has included refurbishment of government housing and school fees to allow staff to send their children away for better education in the retention package [9]. However, little is know at this stage about the long-term impact of the scheme.

## Discussion

If the staffing of rural and remote areas is to be improved, strategies employed by government structures have to address the factors which impact on attraction and retention in a given context. We have not been able to find evidence in the literature that, having identified the factors impacting on attraction and retention, government employers developed appropriate HRM strategies in direct response to such findings. From the literature review, it is evident that certain strategies, such as targeted recruitment and training as well as incentives and compulsion are frequently reported. However, strategies which address immediate living environments are less commonly described in the literature, even though such strategies would be investments not only for the health sector but for the entire population. Interestingly, the literature also reports little evidence of strategies which address management and working conditions at the work place, although the importance of the immediate working environment on attraction and retention has been identified in numerous studies.

**Figure 4:**
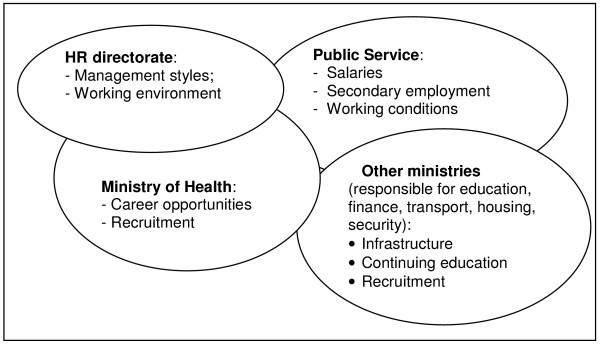
Location of decision-making responsibilities related to HRM and factors related to attraction and retention in the public sector.

What can explain this mismatch? There are likely to be a number of reasons, amongst them the fact that in many countries health planning does not have a good track record of being evidence based. Furthermore, human resource managers often lack the knowledge and skills and sometimes the authority to improve local working conditions in an environment where the political will to address retention issues remains weak. Another possible explanation may lie in the fact that the factors identified fall within the decision-making powers of a number of different government ministries. If mapped onto Figure 3, the location of these decision-making responsibilities becomes clearer (see Figure 4).

The empirical evidence suggests that, with a few exceptions, strategies are not comprehensive and often limited to a single or limited number of factors. If however, as the evidence suggests, "no single intervention is likely to provide a sustainable solution to all workforce challenges facing an organisation [14]," there may be good cause, from a planning perspective, to revisit the way in which strategies are developed, who is involved and how these strategies are coordinated

Firstly, because of the complexity of attraction and retention, there is a strong argument to be made for bundles of interventions. At present there is little documented evidence of such 'bundles' in middle and low income countries. Those exceptions that do exist, such as Thailand and Indonesia, seem to indicate that the introduction of bundles of compulsion and incentives appear to mostly have found favour in periods of political transformation, with the assistance of substantial political backing. More recently Zambia has seen bundles of interventions in their health worker retention scheme which includes financial incentives and career development opportunities in addition to those mentioned earlier.

Secondly, the elements of the bundles needed are diverse (living environment – schools, communications; the job, including career advancement; working conditions), as are the decision-makers in charge of influencing such elements (see Figure 4). Consequently, planning and decision-making to improve retention requires multi-sectoral collaboration within and beyond government. Exactly who should lead and who should be involved in collaboration will vary according to context, but attention to organisational structures such as multi-sectoral task teams and inter-departmental committees would undoubtedly greatly facilitate such collaboration.

Thirdly, there is little evidence in the literature about what really works. More often than not "'how something is done is more important than what is done' – but existing empirical studies concentrate on the latter" (Richard and Thompson as quoted in [14]: 3). Given the complexity of the interventions and the mix of strategies needed, decision makers and managers need to ensure that they monitor and evaluate the impact of the strategies against clear criteria linked to the problem they are trying to assess – and make adjustments where necessary. Furthermore, researchers need to try to gather this data from the operational level to develop case studies so lessons can be shared. This importantly includes country or even location specific variations that need to be understood before developing appropriate strategies.

## Conclusion

The staffing of public sector health facilities in remote rural areas is a serious challenge for many ministries of health. There are HRM strategies for addressing difficulties in attraction and retention, though their effectiveness will depend on a number of steps. First, the analysis of the factors determining attraction and retention. The knowledge about these factors in MLICS is quite well developed. Second, the identification of HRM strategies – usually a bundle of strategies – to respond appropriately to the problems. Much less is known about what individual strategies work in MLICS – and this is further complicated by lack of knowledge about what is the best mix in a bundle. Those are what could be considered the 'technical' steps. The political steps – usually by far the hardest to address – involve bringing the key decision-makers together to form a coherent strategy. An important precursor is to identify who all the key actors are. This paper has provided a simple framework to start that process. What is now needed is more research and further evidence on processes of identification and implementation of HRM strategies to improve attraction and retention, long-term sustainability of such strategies, and the impacts on staffing in remote areas. There are no set answers to the problem of attraction and retention. It is only through learning about what works in terms of fit between problem analysis and strategy and effective navigation through the politics of implementation that any headway will be made against the almost universal challenge of staffing health service in remote rural areas.

## Competing interests

The author(s) declare that they have no competing interests.

## Authors' contributions

UL and TM conceived of the article and had primary responsibility for the initial draft of the manuscript. UL, TM and MD all contributed substantially to the methods, intellectual content of the review, and writing and finalisation of the manuscript. All authors read and approved the final manuscript.

## Pre-publication history

The pre-publication history for this paper can be accessed here:


